# MFCN‐DDI: Capsule network based on multimodal feature for multitype drug–drug interaction prediction

**DOI:** 10.1002/qub2.70021

**Published:** 2025-10-09

**Authors:** Jiayi Lu, Yingying Jiang, Yuhan Fu, Mengdi Nan, Qing Ren, Jie Gao

**Affiliations:** ^1^ School of Science Jiangnan University Wuxi Jiangsu China

**Keywords:** capsule network, drug–drug interaction, edge‐featured graph attention network, multimodal feature

## Abstract

Precise prediction of drug–drug interactions (DDIs) is essential for pharmaceutical research and clinical applications to minimize adverse reactions, optimize therapies, and reduce costs. However, existing methods still face challenges in effectively integrating multidimensional drug features and fully utilizing edge features in molecular graphs, which are crucial for predicting DDIs precisely. Moreover, current methods may not adequately capture the complex relationships between different types of features, limiting predictive performance. This paper proposes the MFCN‐DDI model for DDI type prediction. The model consists of a multimodal feature extraction module, a capsule network‐based feature fusion module, and a DDI predictor module. In the multimodal feature extraction module, four kinds of features are used to provide rich and comprehensive representations for subsequent DDI type prediction, where molecular graph features are generated by considering molecular graphs with edge features. The capsule network‐based feature fusion module captures complex feature relationships to generate high‐quality integrated representations. In the DDI predictor module, multiclass and multilabel classification predictions are performed accurately. Experimental results show that MFCN‐DDI outperforms existing comparison models in prediction tasks. Case studies further prove its practical applicability. In summary, MFCN‐DDI provides an efficient and reliable solution for DDI prediction.

## 1 INTRODUCTION

Drug therapy is a crucial tool in modern medicine, with drug combination therapy now widely adopted for managing complex diseases such as cancer and tuberculosis. This approach improves efficacy and reduces drug resistance by targeting multiple proteins within the same or across pathways [1, 2]. However, the complexity of combining drugs significantly raises the potential for drug–drug interactions (DDIs) [3], where the pharmacological action of one drug is altered by the presence of another [4]. DDIs can lead to reduced efficacy or cause serious adverse reactions, posing a threat to patient health. Thus, predicting potentially harmful DDIs is critical for optimizing treatment regimens and ensuring drug safety. Traditional DDI prediction methods mainly rely on in vivo and in vitro experiments, but the predictive power of such methods is significantly limited by the excessive cost and prolonged duration of these experiments, as well as the wide variety of drug candidates [5]. To overcome these limitations, DDI prediction methods based on computational approaches have emerged as an important aid in biochemical experiments [6]. These computational approaches help reduce risks in clinical trials and improve overall drug effectiveness.

Currently, computational approaches for DDI prediction fall into three major types [7]: the models based on literature extraction [8, 9], the models based on pharmacovigilance data mining [10], and the prediction models based on machine learning [3]. Among them, literature extraction‐based models apply natural language processing methods to obtain DDI information from biomedical texts; pharmacovigilance data mining‐based models mine DDIs from electronic data through statistical methods; and machine learning‐based prediction models predict potential DDIs prior to drug launch by incorporating features such as drug chemical structures and genomic information. The application of these methods enables researchers to more comprehensively assess DDIs, significantly improving the efficiency of drug development. Among the above methods, the machine learning‐based models have gradually become the focus of research because they can derive features from the multidimensional attributes of drugs to achieve DDI prediction and risk warning during the initial phases of drug development. According to the task objectives, the machine learning‐based models are categorized into binary DDI prediction models, which determine whether drugs interact [11], and DDI type prediction models, which predict specific interaction types. Early research on binary DDI prediction has yielded significant results, with various frameworks utilizing machine learning approaches to enhance predictive performance.

After the advancement of deep learning, researchers have shifted focus from predicting whether DDIs occur to predicting the specific types of DDIs, a more complex but practical task [12]. DDI type prediction tasks are categorized into multiclass classification (one DDI type per drug pair) and multilabel classification (multiple DDI types per drug pair) [12, 13]. Models for these tasks fall into three main types: the traditional machine learning‐based models, the deep neural network (DNN)‐based models, and the graph neural network (GNN)‐based models. In traditional machine learning models, Shi et al. [14] developed TMFUF, which used triple matrix decomposition to relate drug features to DDIs and predict whether DDIs were enhanced or weakened. Among the DNN models, DeepDDI developed by Ryu et al. [15] constructed structural similarity vectors based on drug fingerprints and predicted 86 DDI types by applying DNN. Deng et al. [16] proposed DDIMDL, which constructed submodels using four drug similarity matrices with DNNs and combined their outputs via mean operators for DDI prediction. Masumshah et al. [17] introduced DPSP, which extracted drug features using Jaccard similarity and combined them with DNNs to achieve DDI type prediction. Deng et al. [18] designed META‐DDIE, a few‐shot learning method that predicted DDI events by learning drug representations and comparing their similarities. Wang et al. [19] proposed ZeroDDI, which incorporated biological semantics into DDI representation learning and employed a dual‐modal uniform alignment strategy to predict zero‐shot DDI events while addressing category imbalance. In addition, graph neural network‐based models have made important breakthroughs in DDI type prediction. For example, the Decagon proposed by Zitnik et al. [20] transformed DDI multilabel prediction into multirelational link prediction within a multimodal network and used graph convolutional networks for feature extraction. SumGNN developed by Yu et al. [12] combined the knowledge graph (KG) and the multirelational graph convolutional algorithm to achieve accurate predictions. Li et al. [21] designed the DSN‐DDI model to learn drug substructures using inter‐view and intra‐view data concurrently, providing a new solution idea for DDI type prediction. MRCGNN developed by Xiong et al. [22] integrated drug molecular structures and multirelational DDI information, using contrastive learning with dual‐view negative sample augmentation to improve rare DDI event prediction. These studies show that through the application of deep learning methods, especially graph neural networks, researchers can understand the complicated mechanisms of DDIs more comprehensively, thus advancing the field of DDI prediction.

Despite many advances in DDI type prediction, there is still room for improvement. First, existing graph neural network methods mainly focus on node features while paying less attention to edge features when dealing with molecular graphs, although edge features are equally important for understanding DDIs. Second, current methods may not fully capture the potential associations between different types of features. Finally, with the increasing amount of pharmaceutical data, there is a strong need to utilize deep learning techniques for effective integration of diverse data to enhance predictive performance [23]. Therefore, effectively integrating the multidimensional features of drugs to improve prediction accuracy remains an important challenge at present. To overcome these limitations, we design the MFCN‐DDI model, which fully utilizes the edge features of drug molecules to extract molecular graph representations and combines them with KG features, molecular fingerprint features, and pharmacological features of drugs. Furthermore, the model employs a capsule network‐based feature fusion module to capture complex feature relationships for multitype DDI prediction. MFCN‐DDI is evaluated in two prediction tasks, and the importance of every feature and module, as well as its effectiveness and applicability in practice, are verified by ablation studies and case studies. Experimental results indicate that MFCN‐DDI holds benefits toward improving the accuracy of DDI prediction, which can provide strong support for the safe use of clinical medication and guarantee the optimization of drug combination therapy and patient health.

## 2 RESULTS

### 2.1 Setup

To comprehensively assess MFCN‐DDI’s performance for DDI type prediction, the task is categorized into two scenarios: multiclass and multilabel. Correspondingly, we employ a multiclass dataset, a small‐scale multiclass dataset, and a multilabel dataset, splitting all drug pairs into training and test sets. To ensure evaluation reliability, five‐fold cross‐validation is conducted for every task, and final results are obtained by averaging the outcomes of five experiments.

While training, a batch size of 1024 is employed, and model parameters are updated via the Adam optimizer. The learning rate begins at 0.001, with a weight decay factor of 0.0001. To further optimize the training process, a learning rate scheduler reduces the rate to 10% of its previous value every 50 epochs. Additionally, an early stopping mechanism halts training if performance is not advanced over 20 consecutive epochs, saving computational resources and time. The feature vector dimensions, as well as the architectures of the edge‐featured graph attention network (EGAT) and capsule network, are set according to prior studies and experience [24, 25, 26]. These hyperparameters, including the architectures of the multiclass predictor and multilabel predictor, batch size, weight decay factor, learning rate, and early stopping patience, are determined based on extensive empirical testing to achieve optimal convergence and performance.

As for the evaluation metrics, regarding multiclass task, we choose the common metrics, including AUPR (area under the precision‐recall curve), AUC (area under the receiver operating characteristic curve), kappa [27], accuracy, and macro‐precision. Concerning multilabel tasks, AUPR and AUC are the main evaluation metrics employed. The specific descriptions of all evaluation metrics are shown in Section A of Supporting Information S1.

### 2.2 Baseline

In order to fully validate MFCN‐DDI’s performance, we select six classical and widely used baselines. These methods employ drug features such as KGs, molecular fingerprints, molecular graph features, and pharmacological features, either individually or in partial combinations. Most of them utilize graph neural networks or DNNs to extract important drug representations for DDI type prediction.

- DeepDDI [15] utilized DNN to predict DDI types from the drug structure similarity vector.
- DDIMDL [16] combined multimodal drug features and constructed cross‐modal representations to efficiently predict DDI types by fusing deep learning submodels with a joint DNN architecture.
- MUFFIN [13] introduced a KG to fuse knowledge embedding with structural features of drug molecules to predict DDI types.
- MDF‐SA‐DDI [28] was a new method based on multisource drug fusion and the transformer self‐attention mechanism to predict DDI types, which enhanced the prediction accuracy by fusing drug features.
- MSEDDI [29] integrated multiscale embeddings from biomedical KGs, simplified molecular input line entry system (SMILES) sequences, and molecular graphs using a three‐channel architecture with self‐attention‐based feature fusion to effectively predict DDI types.
- MCFF‐MTDDI [24] obtained KG features, extra label information, and drug chemical structure features, and effectively integrated different feature representations through a multichannel feature fusion module.

### 2.3 Comparison results

To comprehensively evaluate MFCN‐DDI’s performance, we conduct performance tests of MFCN‐DDI on a multiclass dataset, a multilabel dataset, and a small‐scale multiclass dataset. Meanwhile, we conduct comparison experiments between the proposed model and six baseline models to verify its superiority. Tables 1 and 2 present in detail the performance comparison results between MFCN‐DDI and the six baselines for the multiclass task and multilabel task.

**TABLE 1:** Performance comparison results of MFCN‐DDI with baseline models on a multiclass dataset and a multilabel dataset.

Methods	Multiclass dataset					Multilabel dataset	
	AUPR	AUC	Kappa	Accuracy	Macro‐precision	AUPR	AUC
DeepDDI	0.8607	0.9888	0.8734	0.8937	0.8581	0.5872	0.8808
DDIMDL	0.8406	0.9872	0.9460	0.9546	0.9185	0.6664	0.9101
MUFFIN	0.9423	0.9883	0.9433	0.9524	0.9173	0.6732	0.9120
MDF‐SA‐DDI	0.8947	0.9870	0.9017	0.9170	0.8121	0.6420	0.8966
MSEDDI	0.9338	0.9887	0.9335	0.9440	0.9035	0.6390	0.8929
MCFF‐MTDDI	0.9465	0.9884	0.9592	0.9657	0.9326	0.6770	0.9168
**MFCN‐DDI**	**0.9485**	**0.9895**	**0.9688**	**0.9738**	**0.9417**	**0.6878**	**0.9191**

Abbreviations: AUC, area under the receiver operating characteristic curve; AUPR, area under the precision‐recall curve; DDI, drug–drug interaction.

*Note:* The best results are highlighted in bold.

**TABLE 2:** Performance comparison results of MFCN‐DDI with baseline models on a small‐scale multiclass dataset.

Methods	Small‐scale multiclass dataset				
	AUPR	AUC	Kappa	Accuracy	Macro‐precision
DeepDDI	0.7048	0.9827	0.7401	0.7850	0.6963
DDIMDL	0.6257	0.9815	0.8133	0.8458	0.7102
MUFFIN	0.8518	0.9833	0.8656	0.8888	0.8049
MDF‐SA‐DDI	0.7979	0.9885	0.8421	0.8690	0.7350
MSEDDI	0.7842	**0.9896**	0.8146	0.8459	0.6952
MCFF‐MTDDI	0.8408	0.9867	0.8633	0.8868	0.7841
**MFCN‐DDI**	**0.8677**	0.9886	**0.8761**	**0.8973**	**0.8382**

Abbreviations: AUC, area under the receiver operating characteristic curve; AUPR, area under the precision‐recall curve; DDI, drug–drug interaction.

*Note:* The best results are highlighted in bold.

The tables show that MFCN‐DDI delivers excellent performance on several evaluation metrics. In two datasets for the multiclass task, our model reaches 0.9485, 0.9895, 0.9688, 0.9738, and 0.9417 on the AUPR, AUC, kappa, accuracy, and macro‐precision metrics, respectively, on the multiclass dataset, which are better than the other models, reflecting the strong generalization capability in the multiclass task and indicating that the model has high robustness in distinguishing different categories. On the small‐scale multiclass dataset, although our model slightly underperforms the best model in terms of AUC with a score of 0.9886, it still maintains excellent performance and achieves the best results in the other four evaluation metrics. This overall performance fully demonstrates the stability and reliability of the model on the small‐scale dataset. In the multilabel task, our model also performs well. It reaches 0.9191 and 0.6878 in the AUC and AUPR metrics, which are better than the other baseline models, demonstrating the model’s strong competitiveness in the multilabel prediction scenario.

Taken together, our model is able to effectively fuse multimodal features in these two tasks, showing strong prediction ability and generalization performance, providing a more advantageous solution for DDI type prediction.

### 2.4 Ablation studies

For assessing each module’s role in MFCN‐DDI’s prediction performance, we design ablation studies to specifically compare the full model with multiple variants on two tasks (as shown in Tables 3 and 4). The design and purpose of these variant models are as follows:

- MFCN‐DDI‐WKG: A variant with the KG feature removed, which is used to assess its effect on performance.
- MFCN‐DDI‐WFP: A variant with the molecular fingerprint feature removed, which is used to assess its effect on performance.
- MFCN‐DDI‐WMG: A variant with the molecular graph feature removed, which is used to assess its effect on performance.
- MFCN‐DDI‐WPH: A variant with the pharmacological feature removed, which is used to assess its effect on performance.
- MFCN‐DDI‐WEGAT: A variant for extracting the drug molecular graph feature using graph attention network (GAT) instead of EGAT, which is used to evaluate whether using EGAT to extract the molecular graph feature helps to improve prediction performance.
- MFCN‐DDI‐WCAPSULE: The capsule network‐based feature fusion module is removed and replaced by a variant that obtains the comprehensive feature of the drug pairs by directly splicing the KG feature, molecular fingerprint feature, molecular graph feature, and pharmacological feature of the drug pairs, aiming at exploring the enhancement effect of the capsule network on feature fusion.

**TABLE 3:** Performance comparison results of MFCN‐DDI with the variants of MFCN‐DDI on a multiclass dataset and a multilabel dataset.

Methods	Multiclass dataset					Multilabel dataset	
	AUPR	AUC	Kappa	Accuracy	Macro‐precision	AUPR	AUC
MFCN‐DDI‐WKG	0.9435	**0.9897**	0.9590	0.9656	0.9369	0.6725	0.9135
MFCN‐DDI‐WFP	0.9348	0.9863	0.9607	0.9670	0.9190	0.6621	0.9093
MFCN‐DDI‐WMG	0.9443	0.9894	0.9642	0.9699	0.9313	0.6724	0.9126
MFCN‐DDI‐WPH	0.9403	0.9853	0.9624	0.9684	0.9312	0.6569	0.9060
MFCN‐DDI‐WEGAT	0.9386	0.9876	0.9631	0.9690	0.9395	0.6623	0.9080
MFCN‐DDI‐WCAPSULE	0.9460	0.9891	0.9513	0.9591	0.9369	0.6758	0.9159
**MFCN‐DDI**	**0.9485**	0.9895	**0.9688**	**0.9738**	**0.9417**	**0.6878**	**0.9191**

Abbreviations: AUC, area under the receiver operating characteristic curve; AUPR, area under the precision‐recall curve; DDI, drug–drug interaction.

*Note:* The best results are highlighted in bold.

**TABLE 4:** Performance comparison results of MFCN‐DDI with the variants of MFCN‐DDI on a small‐scale multiclass dataset.

Methods	Small‐scale multiclass dataset				
	AUPR	AUC	Kappa	Accuracy	Macro‐precision
MFCN‐DDI‐WKG	0.8380	0.9834	0.8550	0.8796	0.8136
MFCN‐DDI‐WFP	0.8031	0.9852	0.8428	0.8698	0.7646
MFCN‐DDI‐WMG	0.7865	0.9857	0.8369	0.8650	0.7519
MFCN‐DDI‐WPH	0.7776	0.9785	0.8375	0.8655	0.7732
MFCN‐DDI‐WEGAT	0.7728	0.9801	0.8344	0.8628	0.7618
MFCN‐DDI‐WCAPSULE	0.8436	0.9879	0.8571	0.8816	0.8068
**MFCN‐DDI**	**0.8677**	**0.9886**	**0.8761**	**0.8973**	**0.8382**

Abbreviations: AUC, area under the receiver operating characteristic curve; AUPR, area under the precision‐recall curve; DDI, drug–drug interaction.

*Note:* The best results are highlighted in bold.

Tables 3 and 4 show that each variant performs weaker than the full model to varying degrees, which fully illustrates the importance of each module and feature for classification accuracy. In the multiclass task, on the multiclass dataset, after removing the KG feature, although the AUC is slightly higher than that of the full model, macro‐precision, accuracy, kappa, and AUPR decrease, indicating that the KG feature still has a positive effect on overall performance; removing the molecular fingerprint feature results in a notable decrease in model performance, with macro‐precision, kappa, and AUPR decreasing by 2.41%, 0.84%, and 1.44%, respectively; removing the molecular graph feature results in a decrease of 1.10% for macro‐precision; removing the pharmacological feature results in a decrease of 1.12%, 0.66%, and 0.86% for macro‐precision, kappa, and AUPR, respectively; after replacing EGAT with GAT, all the model metrics decrease, especially AUPR, which decreases by 1.04%; removing the capsule network module also results in a decrease in all model metrics, especially kappa, which decreases by 1.81%, indicating that the capsule network is of significant value in feature fusion for model performance improvement. On the small‐scale multiclass dataset, the full model outperforms all its variants in every evaluation metric, demonstrating its stable and superior performance on smaller datasets. Furthermore, we compare the AUC and AUPR scores of the full model and its variants for some DDI types with fewer than 30 samples in both datasets. The results are shown in Tables S2–S5, which indicate that the full model still achieves the best performance, highlighting its stronger ability to handle imbalanced data. In the multilabel task, each variant underperforms the full model, further confirming the full model’s advantage in handling this task.

Overall, for both prediction tasks, the complete model attains the best or near‐best outcomes across all metrics. This suggests that the complementary effect of the KG feature, molecular fingerprint feature, molecular graph feature, and pharmacological feature, as well as the EGAT‐based molecular graph feature extraction and capsule network‐based feature fusion module, are important to enhance model performance. These results further exhibit the strength and reliability of the full model in DDI type prediction tasks.

### 2.5 Case studies

To evaluate the reliability of MFCN‐DDI in real‐world applications, case studies are designed in this section to analyze it in depth. We use all DDIs in the multiclass dataset as a training set and predict drug pairs outside the dataset after training the prediction model. The studies extract the 15 types with the highest number of DDIs in the multiclass dataset and predict scores for potential drug combinations for each type. Ultimately, we select the highest‐ranked 20 DDIs from each type with the highest prediction scores and validate them using DrugBank [30] and the Drug Interactions Checker tool on the Drugs website.

In the analysis of the results, we find that the distribution of prediction scores for five of the 15 DDI types is highly concentrated, with over 20 DDIs scoring 1, and it is not possible to definitively select the highest‐ranked 20 DDIs. Therefore, this work provides detailed discussion and analysis of the predicted outcomes for the remaining 10 DDI types. Table S6 lists the number of DDIs within the highest‐ranked 20 DDIs in the prediction scores of 10 types that are verified as real by the tool. Of the 200 predicted DDIs of these 10 types, 82 DDIs are confirmed, and the specific prediction results for each type are shown in Tables S7–S16.

Analyzing the prediction results reveals that the predictions of the model for certain DDI types are closely related to specific drugs. For example, in Table S13, most of the predicted drug pairs are associated with the hypoglycemic drugs glisoxepide and gliquidone, some of which are confirmed to have the target DDI type, suggesting that the rest of the drug pairs that are not confirmed and that contain either glisoxepide or gliquidone may also have a potential association of that type. In Table S16, most of the drug pairs confirmed are associated with hexoprenaline, further supporting the idea that hexoprenaline may increase hypokalemic activity to some extent. This further confirms the effectiveness of MFCN‐DDI for predicting potential DDIs and provides strong support for discovering drug pairs that may have clinical significance.

Moreover, analyzing the incorrectly predicted drug pairs shows that their actual DDI types are usually correlated with the target DDI types. For example, in Table S11, the 4th and 7th incorrectly predicted DDIs are associated with “the risk or severity of hypertension can be increased,” which is similar to the effect of “decrease the antihypertensive activities.” In Table S15, the types of DDI that are incorrectly predicted focus on an increased risk of hyperkalemia, and it is hypothesized that hyperkalemia and bradycardia may be intrinsically linked. Studies in the literature suggest that hyperkalemia may trigger bradycardias and tachyarrhythmias [31]. In summary, the model is able to learn the intrinsic mechanism of DDI, which provides a valuable reference for the in‐depth study of DDI and screening of drug candidates.

## 3 DISCUSSION AND CONCLUSION

Accurately predicting different DDI types continues to be a major challenge in the task of DDI prediction. In the first place, existing graph neural network methods mainly focus on node features when processing molecular graphs and less on edge features, which are also crucial for the comprehensive understanding of DDIs. Furthermore, current prediction methods have not yet fully explored the potential associations between different features, resulting in the inability to effectively capture complex DDIs. Therefore, effectively fusing multidimensional drug features to enhance prediction accuracy remains a major challenge in current research.

**FIGURE 1:**
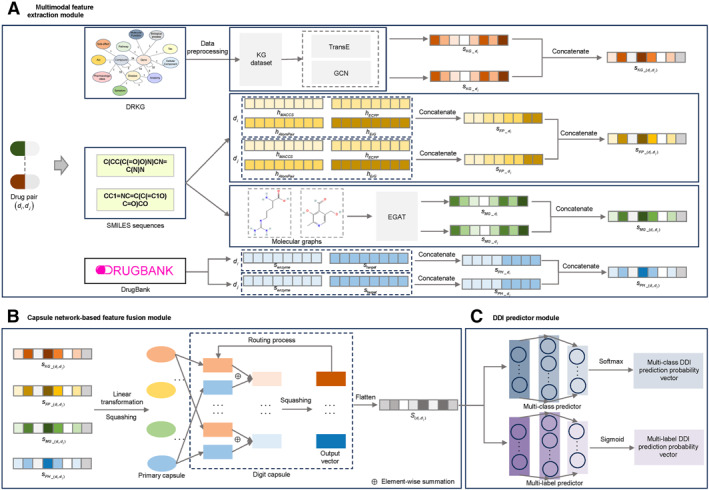
Overview of MFCN‐DDI. (A) Multimodal feature extraction module: This module extracts the KG feature $s_{\text{KG}\_\left(d_{i},d_{j}\right)}$, molecular fingerprint feature $s_{\text{FP}\_\left(d_{i},d_{j}\right)}$, molecular graph feature $s_{\text{MG}\_\left(d_{i},d_{j}\right)}$, and pharmacological feature $s_{\text{PH}\_\left(d_{i},d_{j}\right)}$ of drug pairs. (B) Capsule network‐based feature fusion module: The capsule network fuses multimodal features to construct the comprehensive feature representation $S_{\left(d_{i},d_{j}\right)}$ of drug pairs. (C) DDI predictor module: DDI type classification is achieved using multiclass and multilabel predictors. The molecular graphs and SMILES sequences are obtained from the PubChem database. DDI, drug–drug interaction; SMILES, simplified molecular input line entry system.

**FIGURE 2:**
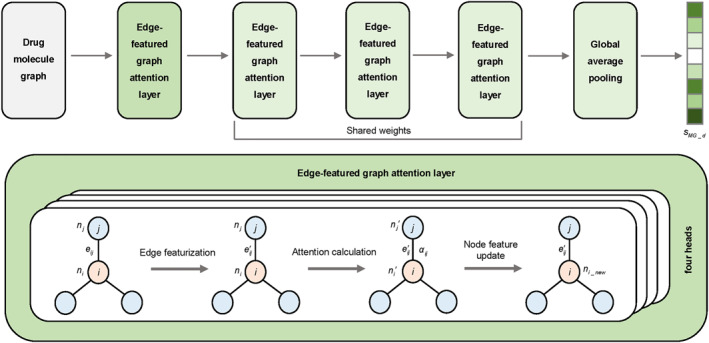
Edge‐featured graph attention network structure diagram.

For overcoming these issues, this work proposes the MFCN‐DDI to accurately predict DDI types. The model framework, shown in Figure 1, contains a multimodal feature extraction module, a capsule network‐based feature fusion module, and a DDI predictor module, significantly enhancing the precision and practicality of DDI prediction. Through the multimodal feature extraction module, the model is able to comprehensively characterize the multidimensional properties of drugs, including the KG features extracted from the KG, the molecular fingerprint features generated based on SMILES sequences, the molecular graph features extracted by the EGAT method (see Figure 2 for the EGAT method flow), and the pharmacological features containing enzyme and target information. Especially in molecular graph feature extraction, the EGAT method fully considers edge features and enhances the integrity of molecular structure expression. In the capsule network‐based feature fusion module, the capsule network‐based fusion strategy can effectively capture the complex relationship between different features to generate more comprehensive and expressive feature representations. The DDI predictor module achieves accurate prediction of DDI types for multiclass and multilabel tasks.

We apply MFCN‐DDI to three constructed datasets (multiclass dataset, multilabel dataset, and small‐scale multiclass dataset) to verify its excellent performance in these two tasks and compare it with six other models. The results of Tables 1 and 2 show that MFCN‐DDI achieves significant prediction results and exhibits strong prediction capabilities on both these tasks. Furthermore, ablation study results (see Tables 3, 4, and S2–S5) show that the synergistic effect of drug KG features, molecular fingerprint features, molecular graph features, and pharmacological features, as well as the EGAT‐based molecular graph feature extraction and capsule network‐based feature fusion module, are crucial to boosting model performance, further highlighting the strengths of the complete model for DDI type prediction tasks. Moreover, case studies are conducted using the multiclass dataset to predict drug pairs outside of the dataset, and Tables S6–S16 demonstrate specific validation results, further proving the model’s practical utility. Meanwhile, the analysis reveals that the model has a strong correlation with specific drugs in predicting certain DDI types, and the actual DDI types of incorrectly predicted drug pairs are usually potentially correlated with the target types.

In conclusion, MFCN‐DDI offers an innovative and efficient approach to multitype DDI prediction, improves accuracy by fusing a variety of drug features with the powerful expressive ability of capsule networks, and provides strong support for DDI prediction in pharmaceutical research and medical practice. In future work, we plan to enhance the interpretability of our framework to facilitate clinical understanding of predictions, explore richer drug features and feature learning methods for more comprehensive representations, and extend the evaluation to include more types of DDIs and broader clinical datasets to further improve prediction utility and generalization.

## 4 MATERIALS AND METHODS

### 4.1 Dataset description

#### 4.1.1 KG dataset

We use the drug repurposing knowledge graph (DRKG) [32] to construct the dataset of the biomedical KG, which contains 97,238 entities categorized into 13 entity types and 5,874,261 triples associated with 107 edge types. In order to avoid introducing additional DDI prior information into the prediction model, we first remove all edges in the DRKG that contain the DDI information. This operation results in 469 drug nodes becoming isolated nodes. We then remove these isolated nodes and use the remaining triples as the final KG dataset for subsequent analysis.

#### 4.1.2 DDI datasets

To comprehensively assess the performance of MFCN‐DDI, three specialized DDI datasets are designed: multiclass dataset and small‐scale multiclass dataset for the multiclass classification task, and multilabel dataset for the multilabel classification task. As a result, three different DDI datasets are obtained.

- Multiclass dataset. We select the dataset of DeepDDI [15] as the data source for multiclass DDIs. This dataset contains 191,878 drug pairs from 86 DDI types, totaling 192,284 DDIs. First, more than 99.8% of the drug pairs in this dataset have a single DDI type, and we delete the minority (0.2%) to ensure that each drug pair is uniquely categorized by one DDI type in this dataset. Then, the processed DeepDDI [15] dataset is intersected with the KG dataset by taking the intersection and removing the drugs for which no SMILES sequences are collected, as well as the drugs for which molecular graphs could not be constructed by SMILES, to obtain the multiclass dataset, which consists of 189,121 DDIs of 85 DDI types from 1677 drugs.
- Small‐scale multiclass dataset. We select the dataset by Deng et al. [16] as the data source. This dataset is relatively small in scale, with relatively balanced sample sizes across each DDI type, and includes 74,528 DDIs for 572 drugs of 65 DDI types. After removing duplicate drug pair interactions, the dataset contains 37,264 DDIs. We then perform the same operations as when constructing the multiclass dataset to obtain the small‐scale multiclass dataset, which consists of 35,690 DDIs of 64 DDI types from 551 drugs.
- Multilabel dataset. We use the TWOSIDES [33] dataset as the data source for multilabel DDIs, which includes 211,990 DDIs for 1918 drugs of 12,726 DDI types. We first take the intersection of the TWOSIDES [33] dataset with the KG dataset and retain drugs with SMILES sequences. On this basis, to ensure that the size of the dataset is sufficient, we remove types containing less than 10,000 DDIs and select the top 200 DDI types as the predicted types in ascending order of the number of drug pairs. We then remove those drugs for which it is not possible to construct molecular graphs using SMILES sequences to obtain the multilabel dataset, which includes 98,854 DDIs from 1287 drugs of the selected 200 DDI types.

The description of three DDI datasets is presented in Table 5.

**TABLE 5:** Statistics of the datasets.

Dataset	Drugs	DDIs	DDI types
Multiclass dataset	1677	189,121	85
Small‐scale multiclass dataset	551	35,690	64
Multilabel dataset	1287	98,854	200

Abbreviation: DDI, drug–drug interaction.

### 4.2 Overview of MFCN‐DDI

The framework of MFCN‐DDI, presented in Figure 1, mainly includes three modules: multimodal feature extraction module, capsule network‐based feature fusion module, and DDI predictor module, aiming at accurately predicting the types of DDIs. In the multimodal feature extraction module, this model extracts four kinds of features to comprehensively reflect the multidimensional properties of the drugs: (1) KG feature: The drug KG features are extracted from the KG dataset by using TransE [34] and GCN [35]. (2) Molecular fingerprint feature: Molecular fingerprint features of the drugs are extracted in four types based on the SMILES sequences. (3) Molecular graph feature: The SMILES sequences are utilized to generate the molecular graphs of the drugs, and molecular graph features of the drugs are obtained using EGAT [25]. (4) Pharmacological feature: containing drug‐related enzyme and target information. In the capsule network‐based feature fusion module, the capsule network fuses various feature types to capture their relationships and construct the comprehensive feature representation. In the DDI predictor module, the model employs a multiclass predictor and a multilabel predictor to cope with different classification tasks so as to achieve accurate prediction of DDI types.

### 4.3 Multimodal feature extraction module

#### 4.3.1 KG feature

We use $G_{\text{kg}}=\left\{\left(h,r,t\right)|h,t\in E,r\in R\right\}$ as the KG, where *E* represents the set of entities and *R* denotes the set of relations in the KG. For each entity and relation in $G_{\text{kg}}$, we employ TransE [34], a KG embedding approach, for generating the original representation based on the KG. TransE [34] can effectively capture the associations among entities and relations, and provide a high‐quality embedding representation basis for subsequent KG applications. Its core concept is to make the sum of the embedding $v_{h}$ of head entity and the embedding $v_{r}$ of relation as close as possible to the embedding $v_{t}$ of tail entity regarding the triple (h,r,t) in $G_{\text{kg}}$, that is, $v_{h}+v_{r}\approx v_{t}$, where $v_{h},v_{r},v_{t}\in R^{d}$. Its loss function is expressed below, with the objective of minimizing it during training:

(1) $$L_{TransE}=\sum_{\left(h,r,t\right)\in Q}\sum_{\left(h^{\prime },r,t^{\prime }\right)\in Q^{\prime }}{\left[t\left(v_{h},v_{r},v_{t}\right)+\lambda -t\left(v_{h^{\prime }},v_{r},v_{t^{\prime }}\right)\right]}_{+},$$

(2) $$t\left(v_{h},v_{r},v_{t}\right)={\left\|v_{h}+v_{r}-v_{t}\right\|}_{L2},$$

where $Q^{\prime }=\left\{\left(h^{\prime },r,t\right)|h^{\prime }\in E\right\}\cup \left\{\left(h,r,t^{\prime }\right)|t^{\prime }\in E\right\}$, and λ is the margin hyperparameter. *Q* and $Q^{\prime }$ represent the set of positive triples and the set of negative triples, where $Q^{\prime }$ is generated through random substitution of head entities or tail entities of the triples in the set of positive triples *Q* by some other entities. This yields the original KG‐based embedding representation $h_{\text{original}}\in R^{400\times 1}$ of the drug node.

After extracting the original representations of the nodes, in order to sufficiently integrate the information about the neighboring nodes of each node for updating the drug representations, we introduce GCN [35] for message passing. The traditional GCN achieves the aggregation of neighboring node features through the normalization of the adjacency matrix, but to more reasonably portray the influence weights between nodes, we adapt the traditional GCN to more comprehensively reflect the structural features of nodes and their neighborhoods. The node update formula for each layer is given below:

(3) $$h_{i}^{\left(l+1\right)}=\text{ReLU}\left(\sum_{j\in N_{i}}\omega_{ij}W^{\left(l\right)}h_{j}^{\left(l\right)}+\omega_{i}h_{i}^{\left(l\right)}\right),$$

where $\omega_{ij}=1/\sqrt{\left|N_{i}\right|\left|N_{j}\right|}$, $\omega_{i}=1/\left|N_{i}\right|$, $N_{i}$, and $N_{j}$, respectively, denote the neighborhoods of node *i* and *j*; $W^{\left(l\right)}$ denotes the layer *l* trainable parameters; and $h_{i}^{\left(l\right)}$ and $h_{j}^{\left(l\right)}$ represent the features of node *i* and *j* in layer *l*, respectively. The model uses the original KG‐based embedding representations as the original features of each node. Finally, the final output node features of the model are normalized to generate KG feature $s_{\text{KG}\_d}\in R^{400\times 1}$ for drug *d*. The KG feature $s_{\text{KG}\_\left(d_{i},d_{j}\right)}\in R^{800\times 1}$ of a drug pair $\left(d_{i},d_{j}\right)$ is generated through concatenating KG features of two drugs in this pair. The detailed process for this section is shown in Algorithm S1.

#### 4.3.2 Molecular fingerprint feature

Molecular fingerprint is a numerical representation for describing the structure of chemical molecules, which makes it an important tool for studying intermolecular interactions by converting complex molecular structures into easy‐to‐handle fixed‐length vectors [36]. In DDI prediction, the molecular fingerprint feature can reveal the potential relationship between drugs from the perspective of molecular structure, thus providing key structural information to support the model. Four commonly used types of molecular fingerprints are selected for this research: the molecular access system (MACCS) fingerprint [37], the extended connectivity fingerprint (ECFP) [38] with a maximum diameter parameter of four, the AtomPair fingerprint [39], and the pharmacophore ErG fingerprint [40]. The MACCS fingerprint [37] is a dictionary‐based molecular fingerprint that describes molecular features in the form of binary coding used to signify whether or not a molecule contains specific structural fragments or functional groups, which is commonly used to characterize molecules for drug activity, toxicity, interactions, etc. ECFP [38] is a circular fingerprint that records information about the state of atoms or bonds around each feature site by traversing the chemical graph of the molecule, centered on a specific radius and feature site. It is applicable to molecular structure characterization and similarity analysis. AtomPair fingerprint [39] is a topological fingerprint, which characterizes the molecular structure by recording the properties of atomic pairs in the molecule and their spatial distances, and can effectively reflect the three‐dimensional spatial distribution properties of drugs, which helps to study the spatial interactions between drug molecules. ErG [40] is a pharmacophore‐based fingerprint, which encodes the relevant molecular properties based on the pharmacophore in the molecule (e.g., hydrogen‐bonding acceptors, ionic receptors, and hydrophobic regions, etc.) and can effectively characterize the pharmacological properties and biological activities of molecules.

From the SMILES sequence of drug *d*, we compute its MACCS fingerprint vector $h_{\text{MACCS}\_d}\in R^{167\times 1}$, ECFP vector $h_{\text{ECFP}\_d}\in R^{1024\times 1}$, AtomPair fingerprint vector $h_{\text{AtomPair}\_d}\in R^{1024\times 1}$, and pharmacophore ErG fingerprint vector $h_{\text{ErG}\_d}\in R^{441\times 1}$ by using the Python package RDKit. These four fingerprints are combined to generate the molecular fingerprint feature $s_{\text{FP}\_d}\in R^{2656\times 1}$ of drug *d*. Next, the molecular fingerprint feature representation $s_{\text{FP}\_\left(d_{i},d_{j}\right)}\in R^{5312\times 1}$ of a drug pair $\left(d_{i},d_{j}\right)$ is constructed by connecting molecular fingerprint features of two drugs in this pair.

#### 4.3.3 Molecular graph feature

We build the molecular graph of the drug based on its SMILES sequence. During the construction of the molecular graph, we extract 69‐dimensional initial feature vectors $n_{i}$ for each atom node, according to the following aspects: atom type, hybridization type, degree (i.e., number of covalent bonds), number of connected hydrogens, implicit valence, whether it belongs to a ring system, and whether it belongs to an aromatic system; similarly, the six‐dimensional initial feature vector $e_{ij}$ is generated for each edge based on features such as the type of bond, whether it is conjugated, and whether it is part of a ring. See Table S1 for specific information. Molecular graph features of drugs are constructed by combining these node features with edge features to provide structured information for further prediction.

To deal with molecular graph features, we employ EGAT [25]. Unlike traditional graph neural network methods, EGAT [25] specifically considers the impact of edge features on graph structure learning. Many existing graph neural network methods focus mainly on node features and ignore edge features, which may lead to information loss when dealing with drug molecules that have complex chemical structures. In contrast, EGAT [25] introduces edge features and enhances the contribution of edge features to node information passing through the attention mechanism to capture the structural relationships in molecular graphs more accurately.

This part is formed by four EGAT layers, where the last three EGAT layers share the parameter weights, as shown in Figure 2.

In each EGAT layer, first of all, the edge feature is updated based on the initial features $n_{i}$ and $n_{j}$ of nodes *i* and *j*, along with the initial feature $e_{ij}$ of the edge between them:

(4) $$e_{ij}^{\prime }=W_{e}\left(n_{i}\left\|e_{ij}\right\|n_{j}\right)+b_{e},$$

where ‖ is the concatenate operation, $W_{e}$ is the parameter matrix, and $b_{e}$ is the bias. In the first EGAT layer, $W_{e}\in R^{512\times 144}$, $b_{e}\in R^{512\times 1}$, and $e_{ij}^{\prime }\in R^{512\times 1}$. In the last three EGAT layers, $W_{e}\in R^{512\times 1536}$, and the dimensions of $b_{e}$ and $e_{ij}^{\prime }$ are unchanged at 512×1.

Subsequently, the feature of each node is projected into a vector space of dimension 512×1 by a linear transformation:

(5) $${n_{i}}^{\prime }=W_{n}n_{i}+b_{n},$$

where $W_{n}$ is the parameter matrix and $b_{n}$ is the bias. In the first EGAT layer, $W_{n}\in R^{512\times 69}$, $b_{n}\in R^{512\times 1}$, and ${n_{i}}^{\prime }\in R^{512\times 1}$. In the last three EGAT layers, $W_{n}\in R^{512\times 512}$, and the dimensions of $b_{n}$ and ${n_{i}}^{\prime }$ remain unchanged at 512×1.

Next, in each EGAT layer, information is transferred between nodes through edges, and feature updating is realized by combining nonlinear transformation. The updated edge feature $e_{ij}^{\prime }$ is converted into an attention score, which quantifies the weight of information passing among the nodes in each attention head. Each layer contains four attention heads, so the node feature ${n_{i}}^{\prime }\in R^{512\times 1}$ is reshaped into the tensor $n_{i,h}^{\prime }\in R^{128\times 1\times 4}$. Each reshaped tensor is weighted and fused with the corresponding tensor $n_{j,h}^{\prime }\in R^{128\times 1\times 4}$ of the neighboring node according to the attention value $\alpha_{ij}\in R^{1\times 1\times 4}$ of the corresponding attention head. These steps are presented thoroughly below.

The edge feature $e_{ij}^{\prime }$ is reshaped into a tensor $e_{ij,h}^{\prime }\in R^{128\times 1\times 4}$. Subsequently, the LeakyReLU function is applied to this tensor to perform a nonlinear transformation and a linear mapping in combination with the parameter tensor *W* in order to compute the attention coefficients between nodes:

(6) $$\varepsilon_{ij}=W\;\text{LeakyReLU}\left(e_{ij,h}^{\prime }\right),$$

where $W\in R^{1\times 128\times 4}$ is the parameter tensor and $\varepsilon_{ij}\in R^{1\times 1\times 4}$. The values $\varepsilon_{ij}$ of all neighboring edges computed by each attention head are then softmax normalized to generate an attention score $\alpha_{ij}$ for each edge:

(7) $$\alpha_{ij}=\text{softmax}\left(\varepsilon_{ij}\right)=\frac{\exp\left(\varepsilon_{ij}\right)}{\sum_{k\in N_{i}}\exp\left(\varepsilon_{ik}\right)},$$

where $N_{i}$ refers to the neighborhood of node *i* and $\alpha_{ij}\in R^{1\times 1\times 4}$. The node feature ${n_{i}}^{\prime }\in R^{512\times 1}$ is reshaped into a tensor $n_{i,h}^{\prime }\in R^{128\times 1\times 4}$. Neighboring node features are weighted and summed with their corresponding attention scores to obtain the updated node feature:

(8) $$n_{i\_\text{new},h}=\sum_{k\in N_{i}}\alpha_{ik}n_{k,h}^{\prime },$$

where $n_{i\_\text{new},h}\in R^{128\times 1\times 4}$ is the tensor, which is reshaped into the vector $n_{i\_\text{new}}\in R^{512\times 1}$ before being output as the layer. Each EGAT layer outputs the updated node features $n_{i\_\text{new}}$ and edge features $e_{ij}^{\prime }$, which will serve as inputs to the next layer.

Finally, with global average pooling operation on all the node features produced from the final EGAT layer, the molecular graph feature vector of the drug, $s_{\text{MG}\_d}\in R^{512\times 1}$, is obtained. The molecular graph feature $s_{\text{MG}\_\left(d_{i},d_{j}\right)}\in R^{1024\times 1}$ of a drug pair $\left(d_{i},d_{j}\right)$ is extracted through concatenating molecular graph features of two drugs in this pair. The detailed process for this section is shown in Algorithm S2.

#### 4.3.4 Pharmacological feature

Pharmacological features are important characterization indicators to reveal the potential interaction mechanisms between drugs and can reflect the interactions between drugs at the molecular level. In this study, two types of binary feature vectors are constructed for each drug based on the DrugBank [30] database: drug‐enzyme feature and drug‐target feature. Among them, each element of the drug‐enzyme feature vector indicates whether the drug has a pharmacological interaction with a specific metabolizing enzyme, and the drug‐target feature vector characterizes whether the drug has a pharmacological interaction with a specific target protein, both of which are coded in a binary way (1 indicates the existence of an interaction, and 0 indicates the absence of one).

About drug *d*, we splice its drug‐enzyme feature $s_{\text{enzyme}\_d}\in R^{344\times 1}$ and drug‐target feature $s_{\text{target}\_d}\in R^{1815\times 1}$ to generate pharmacological feature $s_{\text{PH}\_d}\in R^{2159\times 1}$. The pharmacological feature $s_{\text{PH}\_\left(d_{i},d_{j}\right)}\in R^{4318\times 1}$ of a drug pair $\left(d_{i},d_{j}\right)$ is extracted through concatenating pharmacological features of two drugs in this pair.

### 4.4 Capsule network‐based feature fusion module

For overcoming position information loss that may be caused by the pooling layer in traditional convolutional neural networks, Sabour et al. [41] developed a capsule network. The capsule network is a neural network model capable of capturing spatial relationships and structured information between features and has the advantage over traditional neural networks of being able to better preserve the rich representation of the input features, as well as having strong noise immunity and generalization performance. A dynamic routing algorithm is used here, which can flexibly adjust the weights of the information passing by relevance and contribution of the input features to enhance model performance. Inspired by the feature fusion capabilities of capsule networks in peptide therapeutic property prediction [26], we build on stacked capsule networks to aggregate the extracted multimodal features of drug pairs. In the following, we describe the computational process of this module.

First, based on the four drug pair features obtained earlier, we define a group of feature vectors $s_{\text{modality}\_\left(d_{i},d_{j}\right)}=\left\{s_{\text{KG}\_\left(d_{i},d_{j}\right)},s_{\text{FP}\_\left(d_{i},d_{j}\right)},s_{\text{MG}\_\left(d_{i},d_{j}\right)},s_{\text{PH}\_\left(d_{i},d_{j}\right)}\right\}$ to reflect the multimodal features of a drug pair $\left(d_{i},d_{j}\right)$. Because of the inconsistent dimensions of the vectors in $s_{\text{modality}}$, we feed these multimodal feature vectors into fully connected layers to make their dimensions consistent:

(9) $$S_{\text{modality}}=W_{\text{transform}}\cdot s_{\text{modality}},$$

where $W_{\text{transform}}$ is a set of transformation matrices projecting four multidimensional feature vectors into a 512‐dimensional space.

Next, we use stacked capsule networks to implement the aggregation of features. In the capsule network, features are represented as capsules and propagated through the network. Specifically, we take a set of initial capsules C° as the input to the capsule network, the number of which is the same as the number of features. The initial capsule is computed as shown in the following equation:

(10) $$c_{i}^{0}=\text{Squashing}\left(s_{i}\right)=\frac{{\left\|s_{i}\right\|}^{2}}{1+{\left\|s_{i}\right\|}^{2}}\frac{s_{i}}{\left\|s_{i}\right\|},$$

where $s_{i}\in S_{\text{modality}}$, Squashing() is an activation function, $c_{i}^{0}$ signifies the initial capsule *i* in C°. Regarding the output capsule $c_{j}^{1}$ from the network layer, each initial capsule generates a predictive representation $a_{ij}^{0}$ by multiplying it with the parameter matrix $W_{ij}$:

(11) $$a_{ij}^{0}=W_{ij}^{0}c_{i}^{0}\;.$$

Subsequently, by calculating all predictive representations through weighted summation and applying the routing mechanism, we obtain the set $C^{1}$ of output capsules of the network layer. The computational procedure is defined as follows:

(12) $$f_{j}^{0}=\sum_{i}\beta_{ij}^{0}a_{ij}^{0},$$

(13) $$c_{j}^{1}=\text{Squashing}\left(f_{j}^{0}\right),$$

where $\beta_{ij}^{0}$ is the weight coefficient of the $a_{ij}^{0}$, and its calculation is as follows:

(14) $$\beta_{ij}^{0}=\frac{\exp\left(m_{ij}^{0}\right)}{\sum_{k}\exp\left(m_{ik}^{0}\right)},$$

where $m_{ij}^{0}$ represents the prior probability and is initialized to 0. The capsule network updates $m_{ij}^{0}$ using the output capsule $c_{j}^{1}$ and its representation $a_{ij}^{0}$:

(15) $$m_{ij}^{0}=m_{ij}^{0}+a_{ij}^{0}c_{j}^{1}\;.$$

Here, we construct two‐layer capsule networks.

Finally, we flatten all the final output capsules and connect them together to form a comprehensive feature representation $S_{\left(d_{i},d_{j}\right)}\in R^{1024\times 1}$ of drug pairs. The detailed process for this section is shown in Algorithm S3.

### 4.5 DDI predictor module

In the DDI predictor module, we design a multiclass predictor and a multilabel predictor to cope with different classification tasks, respectively. Both DDI predictors utilize a three‐layer fully connected network architecture, which ensures the model’s efficiency and accuracy in processing complex DDI data. Specifically, the first fully connected layer is equipped with 2048 neurons. Immediately after that, the second fully connected layer is extended to 4096 neurons to further deepen the feature extraction. The last fully connected layer then sets its corresponding neuron count based on different task requirements: For the multiclass predictor, we set 85 and 64 neurons to correspond to 85 and 64 different DDI types, respectively, based on different datasets, whereas for the multilabel predictor, 200 neurons are set. Ultimately, the module outputs a vector $q\left(d_{i},d_{j}\right)$ of multitype DDI prediction probabilities for drug pair $\left(d_{i},d_{j}\right)$. Each dimension in this vector corresponds to the predicted probability of one DDI type. $q\left(d_{i},d_{j}\right)$ is calculated with the following formula:

(16) $$q\left(d_{i},d_{j}\right)=\sigma \left(W_{3}\left(\text{Re}\mathrm{LU}\left(W_{2}\left(\text{Re}LU\left(W_{1}S_{\left(d_{i},d_{j}\right)}+b_{1}\right)\right)+b_{2}\right)\right)+b_{3}\right),$$

where $W_{1}$, $W_{2}$, and $W_{3}$ are the weight matrices; $b_{1}$, $b_{2}$, and $b_{3}$ are the biases. σ is differentiated according to the type of task. For the multiclass predictor, σ uses the softmax function, whereas for the multilabel predictor, σ uses the sigmoid function.

### 4.6 Loss function

Multiclass and multilabel tasks utilize separate loss functions. The multiclass task employs the cross‐entropy loss $l_{\text{mc}}$, calculated as follows:

(17) $$l_{\text{mc}}=-\sum_{c=1}^{N_{c}}z_{c}\;\log\left({\hat{z}}_{c}\right),$$

where $N_{c}$ represents the total DDI types, with $N_{c}=85$ for the multiclass dataset and $N_{c}=64$ for the small‐scale multiclass dataset. $z_{c}\in \left\{0,1\right\}$ is an indicator function for whether or not type *c* is the true label of the drug pair $\left(d_{i},d_{j}\right)$, and ${\hat{z}}_{c}$ is the probability of the drug pair belonging to type *c* as predicted by the model.

For the multilabel task, because each drug pair may belong to more than one type at the same time, the binary cross‐entropy loss $l_{\text{ml}}$ is employed, described as follows:

(18) $$l_{\text{ml}}=-\frac{1}{N_{c}}\sum_{c=1}^{N_{c}}\left[p_{c}z_{c}\;\log\left({\hat{z}}_{c}\right)+\left(1-z_{c}\right)\log\left(1-{\hat{z}}_{c}\right)\right],$$

where $N_{c}=200$ represents the total DDI types in the multilabel dataset; $p_{c}$ is the weight coefficient for positive samples of type *c*, and its value equals the proportion of negative to positive samples [24, 42]. The meanings denoted by $z_{c}$ and ${\hat{z}}_{c}$ are consistent with those in $l_{\text{mc}}$.

Ultimately, for all drug pairs, the total loss function is calculated:

(19) $$L_{\text{total}}=\frac{1}{N}\sum l_{\text{loss}},$$

where *N* represents the total drug pairs. Depending on the task type, $l_{\text{loss}}$ is chosen as either $l_{\text{mc}}$ or $l_{\text{ml}}$.

## AUTHOR CONTRIBUTIONS

**Jiayi Lu**: Conceptualization; methodology; software; visualization; writing—original draft. **Yingying Jiang**: Software. **Yuhan Fu**: Investigation. **Mengdi Nan**: Data curation; validation. **Qing Ren**: Formal analysis; visualization. **Jie Gao**: Funding acquisition; supervision; writing—review and editing.

## CONFLICT OF INTEREST STATEMENT

The authors declare no conflicts of interest.

## ETHICS STATEMENT

This article does not contain any studies with human or animal materials performed by any of the authors.

## Supporting information

Supporting Information S1

## Data Availability

All data employed in this study are from public resources: the dataset of DeepDDI, TWOSIDES, the dataset by Deng et al. is available on the GitHub website (YifanDengWHU/DDIMDL), and DRKG is available on the GitHub website (gnn4dr/DRKG/). The code of MFCN‐DDI is available on the GitHub website (JiayiLu903/MFCN‐DDI/tree/master).
