# Supplementary material for: MFCN‐DDI: Capsule network based on multimodal feature for multitype drug–drug interaction prediction
Source: Quant Biol. 2025 Oct 9;14(1):e70021. doi: 10.1002/qub2.70021 (PMC12806087; doi:10.1002/qub2.70021)
Supplement: Supplementary file 1 — Supporting Information S1 [file QUB2-14-e70021-s001.docx]

**Supplementary Material**

MFCN-DDI: Capsule network based on multimodal feature for multi-type drug–drug interaction prediction

Jiayi Lu

School of Science, Jiangnan University,
Wuxi, Jiangsu, 214122, China
[6231204008@stu.jiangnan.edu.cn](mailto:6231204008@stu.jiangnan.edu.cn)

Yingying Jiang

School of Science, Jiangnan University,
Wuxi, Jiangsu, 214122, China
6231204006@stu.jiangnan.edu.cn

Yuhan Fu

School of Science, Jiangnan University,
Wuxi, Jiangsu, 214122, China
6231204003@stu.jiangnan.edu.cn

Mengdi Nan

School of Science, Jiangnan University,
Wuxi, Jiangsu, 214122, China
6231204011@stu.jiangnan.edu.cn

Qing Ren

School of Science, Jiangnan University,
Wuxi, Jiangsu, 214122, China
6231204012@stu.jiangnan.edu.cn

Jie Gao[[1]](#footnote-1)

School of Science, Jiangnan University,
Wuxi, Jiangsu, 214122, China
[gaojie@jiangnan.edu.cn](mailto:gaojie@jiangnan.edu.cn)

**Section A**

All evaluation metrics are described below:

- AUPR: AUPR denotes the area under the precision-recall (PR) curve with the formula: $AUPR=\sum_{j=1}^{n} PRE_{j}\Delta REC_{j}$, where $PRE_{j}$ and $REC_{j}$ represent the precision and recall at the *j*-th threshold with the formulas $PRE_{j}=\frac{TP^{\left(j\right)}}{TP^{\left(j\right)}+FP^{\left(j\right)}}$ and $REC_{j}=\frac{TP^{\left(j\right)}}{TP^{\left(j\right)}+FN^{\left(j\right)}}$, respectively. The final AUPR value is obtained by averaging the AUPRs of all DDI types.
- AUC: AUC is the area under the receiver operating characteristic curve (ROC) with the equation $AUC=\sum_{j=1}^{n} TPR_{j}\Delta FPR_{j}$, where $TPR_{j}$ and $FPR_{j}$ represent the true positive and false positive rates at the *j*-th threshold with the equations $TPR_{j}=\frac{TP^{\left(j\right)}}{TP^{\left(j\right)}+FN^{\left(j\right)}}$ and $FPR_{j}=\frac{FP^{\left(j\right)}}{FP^{\left(j\right)}+TN^{\left(j\right)}}$, respectively, and $TN^{\left(j\right)}$, $TP^{\left(j\right)}$, $FP^{\left(j\right)}$ and $FN^{\left(j\right)}$ respectively denote the true negative, true positive, false positive and false negative at the *j*-th threshold. We use the average of the AUCs of all DDI types as the final AUC value.
- Kappa: Kappa measures the consistency between predicted classification results and actual classification results. Its formula is: $Kappa=\frac{p_{0}-p_{e}}{1-p_{e}}$,where $p_{0}$ is the observed consistency, i.e., the probability that the predicted classification result is consistent with the actual classification result (the same as the accuracy rate); $p_{e}$ is the random consistency probability, i.e., the probability that the predicted classification result is consistent with the actual classification result, assuming that the classification is completely random.
- Accuracy: Accuracy is the ratio of the number of correctly predicted samples in positive and negative cases to the total number of samples. The formula is: $Accuracy=\frac{1}{N_{c}}\sum_{i=1}^{N_{c}} \frac{TP_{i}+TN_{i}}{TP_{i}+FP_{i}+TN_{i}+FN_{i}}$ , where $N_{c}$ represents the total number of DDI types, and $TN_{i}$, $TP_{i}$, $FP_{i}$ and $FN_{i}$ respectively denote the true negative, true positive, false positive and false negative for DDI type *i*.
- Macro-Precision: Precision is the ratio of correct predictions in a sample of positive predictions. Macro-Precision is obtained by computing the precision rate for each type and averaging the precision rates for all types. Its formula is: $Macro-Precision=\frac{1}{N_{c}}\sum_{i=1}^{N_{c}} \frac{TP_{i}}{TP_{i}+FP_{i}}$.

**Section B**

Table S1.   The atom features and bond features used as the node features and edge features in the drug graph.

| **Name** | **Description** | **Dimension** |
| --- | --- | --- |
| **Atom features** | | |
| Atom type | [C, N, O, S, F, Si, P, Cl, Br, Mg, Na, Ca, Fe, As, Al, I, B, V, K, Tl, Yb, Sb, Sn, Ag, Pd, Co, Se, Ti, Zn, H, Li, Ge, Cu, Au, Ni, Cd, In, Mn, Zr, Cr, Pt, Hg, Pb, other] (one-hot) | 44 |
| Hybridization | [sp, sp2, sp3, sp3d, other] (one-hot) | 5 |
| Degree | The number of covalent bonds [0, 1, 2, 3, 4, 5] (one-hot) | 6 |
| Hydrogen | The number of connected hydrogens [0, 1, 2, 3, 4, 5] (one-hot) | 6 |
| Implicit valence | Implicit valence of the atom [0, 1, 2, 3, 4, 5] (one-hot) | 6 |
| Ring | Whether the atom is part of a ring system [0/1] (binary) | 1 |
| Aromatic | Whether the atom is part of an aromatic system [0/1] (binary) | 1 |
| **Bond features** | | |
| Bond type | [single, double, triple, aromatic] (one-hot) | 4 |
| Conjugate | Whether the bond is conjugate bond [0/1] (binary) | 1 |
| Ring | Whether the bond is part of a ring system [0/1] (binary) | 1 |

Table S2.   AUC scores of MFCN-DDI and its variants for types with few samples on Multi-Class Dataset.

| **Label** | **MFCN-DDI** | **MFCN-DDI-WKG** | **MFCN-DDI-WFP** | **MFCN-DDI-WMG** | **MFCN-DDI-WPH** | **MFCN-DDI-WEGAT** | **MFCN-DDI-WCAPSULE** |
| --- | --- | --- | --- | --- | --- | --- | --- |
| 0 | **1.0000** | 1.0000 | 0.9999 | 1.0000 | 1.0000 | 0.9999 | 1.0000 |
| 30 | **1.0000** | 1.0000 | 1.0000 | 1.0000 | 0.9999 | 1.0000 | 1.0000 |
| 40 | **1.0000** | 1.0000 | 1.0000 | 1.0000 | 0.9999 | 0.9999 | 1.0000 |
| 41 | **1.0000** | 1.0000 | 1.0000 | 1.0000 | 1.0000 | 1.0000 | 1.0000 |
| 42 | **1.0000** | 1.0000 | 0.9999 | 1.0000 | 1.0000 | 0.9999 | 1.0000 |
| 43 | **1.0000** | 1.0000 | 1.0000 | 0.9999 | 0.9999 | 0.9999 | 0.9999 |
| 45 | **1.0000** | 0.9999 | 0.9999 | 0.9999 | 0.9999 | 0.9987 | 0.9999 |
| 49 | **1.0000** | 0.9999 | 0.9999 | 0.9999 | 0.9988 | 0.9986 | 0.9999 |
| 63 | **0.6068** | 0.4606 | 0.3756 | 0.4653 | 0.2499 | 0.4425 | 0.3353 |
| 76 | **1.0000** | 1.0000 | 1.0000 | 1.0000 | 1.0000 | 1.0000 | 1.0000 |

Table S3.   AUC scores of MFCN-DDI and its variants for types with few samples on Small-Scale Multi-Class Dataset.

| **Label** | **MFCN-DDI** | **MFCN-DDI-WKG** | **MFCN-DDI-WFP** | **MFCN-DDI-WMG** | **MFCN-DDI-WPH** | **MFCN-DDI-WEGAT** | **MFCN-DDI-WCAPSULE** |
| --- | --- | --- | --- | --- | --- | --- | --- |
| 46 | **0.9755** | 0.9513 | 0.9441 | 0.9252 | 0.9449 | 0.9595 | 0.9670 |
| 48 | **0.9954** | 0.9817 | 0.9829 | 0.9820 | 0.9432 | 0.9275 | 0.9853 |
| 51 | **1.0000** | 0.9999 | 0.9999 | 0.9999 | 0.9999 | 0.9999 | 0.9999 |
| 53 | **1.0000** | 1.0000 | 0.9998 | 0.9999 | 1.0000 | 0.9998 | 1.0000 |
| 54 | **0.9997** | 0.9855 | 0.9785 | 0.9568 | 0.9743 | 0.9849 | 0.9945 |
| 58 | **0.9999** | 0.9960 | 0.9999 | 0.9934 | 0.9369 | 0.9933 | 0.9913 |
| 59 | **1.0000** | 1.0000 | 1.0000 | 1.0000 | 0.9996 | 0.9999 | 1.0000 |
| 60 | **1.0000** | 1.0000 | 1.0000 | 1.0000 | 1.0000 | 1.0000 | 1.0000 |
| 63 | **0.9999** | 0.8636 | 0.9818 | 0.9095 | 0.8898 | 0.9077 | 0.8247 |

Table S4.   AUPR scores of MFCN-DDI and its variants for types with few samples on Multi-Class Dataset.

| **Label** | **MFCN-DDI** | **MFCN-DDI-WKG** | **MFCN-DDI-WFP** | **MFCN-DDI-WMG** | **MFCN-DDI-WPH** | **MFCN-DDI-WEGAT** | **MFCN-DDI-WCAPSULE** |
| --- | --- | --- | --- | --- | --- | --- | --- |
| 0 | **1.0000** | 1.0000 | 0.9500 | 1.0000 | 1.0000 | 0.9050 | 1.0000 |
| 30 | **1.0000** | 1.0000 | 1.0000 | 1.0000 | 0.9487 | 1.0000 | 1.0000 |
| 40 | **1.0000** | 1.0000 | 1.0000 | 1.0000 | 0.9833 | 0.8807 | 1.0000 |
| 41 | **1.0000** | 1.0000 | 1.0000 | 1.0000 | 1.0000 | 1.0000 | 1.0000 |
| 42 | **1.0000** | 1.0000 | 0.9500 | 1.0000 | 1.0000 | 0.9048 | 1.0000 |
| 43 | **1.0000** | 1.0000 | 1.0000 | 0.9833 | 0.9611 | 0.9417 | 0.9667 |
| 45 | **1.0000** | 0.9933 | 0.9512 | 0.9634 | 0.9933 | 0.9141 | 0.9853 |
| 49 | **0.9952** | 0.9661 | 0.9671 | 0.9521 | 0.9372 | 0.8998 | 0.9933 |
| 63 | **0.0001** | 0.0001 | 0.0000 | 0.0001 | 0.0000 | 0.0001 | 0.0000 |
| 76 | **1.0000** | 1.0000 | 1.0000 | 1.0000 | 1.0000 | 1.0000 | 1.0000 |

Table S5.   AUPR scores of MFCN-DDI and its variants for types with few samples on Small-Scale Multi-Class Dataset.

| **Label** | **MFCN-DDI** | **MFCN-DDI-WKG** | **MFCN-DDI-WFP** | **MFCN-DDI-WMG** | **MFCN-DDI-WPH** | **MFCN-DDI-WEGAT** | **MFCN-DDI-WCAPSULE** |
| --- | --- | --- | --- | --- | --- | --- | --- |
| 46 | **0.7831** | 0.7204 | 0.6698 | 0.7228 | 0.7636 | 0.6895 | 0.7524 |
| 48 | **0.7131** | 0.6798 | 0.5349 | 0.5490 | 0.4203 | 0.4388 | 0.6069 |
| 51 | **1.0000** | 0.9000 | 0.9400 | 0.8952 | 0.9167 | 0.9067 | 0.9667 |
| 53 | **1.0000** | 1.0000 | 0.8722 | 0.9400 | 1.0000 | 0.8800 | 1.0000 |
| 54 | **0.6617** | 0.5678 | 0.4268 | 0.4130 | 0.3375 | 0.4541 | 0.4137 |
| 58 | **0.9667** | 0.7514 | 0.9667 | 0.6375 | 0.5106 | 0.7075 | 0.8006 |
| 59 | **1.0000** | 1.0000 | 1.0000 | 1.0000 | 0.8118 | 0.8667 | 1.0000 |
| 60 | **1.0000** | 1.0000 | 1.0000 | 1.0000 | 1.0000 | 1.0000 | 1.0000 |
| 63 | **0.9000** | 0.8000 | 0.7003 | 0.7001 | 0.6501 | 0.6286 | 0.8000 |

Table S6.   Validation results for the 15 most frequent DDI types.

| **Rank** | **DDI types** | **Known DDIs** | **Confirmed DDIs** | **Details** |
| --- | --- | --- | --- | --- |
| 1 | The risk or severity of adverse effects can be increased when #Drug1 is combined with #Drug2 | 60407 | / | / |
| 2 | The metabolism of #Drug2 can be decreased when combined with #Drug1 | 34149 | / | / |
| 3 | The serum concentration of #Drug2 can be increased when it is combined with #Drug1 | 23405 | / | / |
| 4 | The serum concentration of #Drug2 can be decreased when it is combined with #Drug1 | 9102 | / | / |
| 5 | #Drug1 may increase the hypotensive activities of #Drug2 | 8395 | 3 | Table S7 |
| 6 | The therapeutic efficacy of #Drug2 can be decreased when used in combination with #Drug1 | 7649 | / | / |
| 7 | #Drug1 may increase the QTc-prolonging activities of #Drug2 | 6069 | 5 | Table S8 |
| 8 | #Drug1 may increase the central nervous system depressant (CNS depressant) activities of #Drug2 | 5412 | 6 | Table S9 |
| 9 | The metabolism of #Drug2 can be increased when combined with #Drug1 | 4938 | 10 | Table S10 |
| 10 | #Drug1 may decrease the antihypertensive activities of #Drug2 | 3057 | 5 | Table S11 |
| 11 | #Drug1 may increase the anticoagulant activities of #Drug2 | 3010 | 4 | Table S12 |
| 12 | #Drug1 may increase the hypoglycemic activities of #Drug2 | 2109 | 14 | Table S13 |
| 13 | #Drug1 may decrease the excretion rate of #Drug2 which could result in a higher serum level | 1723 | 6 | Table S14 |
| 14 | #Drug1 may increase the bradycardic activities of #Drug2 | 1277 | 16 | Table S15 |
| 15 | #Drug1 may increase the hypokalemic activities of #Drug2 | 1204 | 13 | Table S16 |

Table S7.   The validation results for the top 20 predicted DDIs of type “#Drug1 may increase hypotensive activities of #Drug2”.

| **Predicted DDIs** | | **Evidence** |
| --- | --- | --- |
| Nicorandil | Choline | Unconfirmed |
| Nicorandil | Dimethyl sulfoxide | Unconfirmed |
| Nicorandil | Edrophonium | Unconfirmed |
| Nicorandil | Acetohydroxamic acid | Unconfirmed |
| Nicorandil | Tipiracil | Unconfirmed |
| Nicorandil | Titanium dioxide | Unconfirmed |
| Nicorandil | Echothiophate | Unconfirmed |
| Nicorandil | Cysteamine | Unconfirmed |
| **Thiopental** | **Nicorandil** | **DrugBank** |
| **Nicorandil** | **Carbidopa** | **DrugBank** |
| Nicorandil | Isoflurophate | Unconfirmed |
| Nicorandil | Technetium Tc-99m sestamibi | Unconfirmed |
| Nicorandil | Pipobroman | Unconfirmed |
| **Aripiprazole** | **Nicorandil** | **DrugBank** |
| Nicorandil | Decamethonium | Unconfirmed |
| Nicorandil | Nitrous oxide | Unconfirmed |
| Thioproperazine | Amifostine | Unconfirmed |
| Nicorandil | Hydroxyurea | Unconfirmed |
| Nicorandil | Zinc oxide | Unconfirmed |
| Nicorandil | Thiabendazole | Unconfirmed |

Table S8.   The validation results for the top 20 predicted DDIs of type “#Drug1 may increase the QTc-prolonging activities of #Drug2”.

| **Predicted DDIs** | | **Evidence** |
| --- | --- | --- |
| Perflutren | Epinephrine | Unconfirmed |
| Perflutren | Phenylephrine | Unconfirmed |
| Zuclopenthixol | Phenylephrine | Unconfirmed |
| Perflutren | Bambuterol | Unconfirmed |
| **Besifloxacin** | **Trazodone** | **DrugBank** |
| Zuclopenthixol | Epinephrine | Unconfirmed |
| Perflutren | Clenbuterol | Unconfirmed |
| **Besifloxacin** | **Bedaquiline** | **DrugBank** |
| Gadobenic acid | Phenylephrine | Unconfirmed |
| Iloperidone | Phenylephrine | Unconfirmed |
| **Crizotinib** | **Benzatropine** | **DrugBank** |
| Bedaquiline | Bambuterol | Unconfirmed |
| Perflutren | Isoprenaline | Unconfirmed |
| Artemether | Phenylephrine | Unconfirmed |
| **Besifloxacin** | **Histrelin** | **DrugBank** |
| Perflutren | Edrophonium | Unconfirmed |
| **Besifloxacin** | **Ofloxacin** | **DrugBank** |
| Flecainide | Epinephrine | Unconfirmed |
| Lenvatinib | Bambuterol | Unconfirmed |
| Ciprofloxacin | Phenylephrine | Unconfirmed |

Table S9.   The validation results for the top 20 predicted DDIs of type “#Drug1 may increase the central nervous system depressant (CNS depressant) activities of #Drug2”.

| **Predicted DDIs** | | **Evidence** |
| --- | --- | --- |
| Ethanol | Dimethyl fumarate | Unconfirmed |
| **Paraldehyde** | **Azatadine** | **Drugs.com** |
| Ethanol | Isothipendyl | Unconfirmed |
| **Paraldehyde** | **Dimetacrine** | **DrugBank** |
| Paraldehyde | Isothipendyl | Unconfirmed |
| **Ethanol** | **Dimetacrine** | **DrugBank** |
| Paraldehyde | Dimethyl fumarate | Unconfirmed |
| Paraldehyde | Carbocisteine | Unconfirmed |
| **Zolpidem** | **Dimetacrine** | **DrugBank** |
| **Methotrimeprazine** | **Dimetacrine** | **DrugBank** |
| Mirtazapine | Isothipendyl | Unconfirmed |
| Zolpidem | Carbocisteine | Unconfirmed |
| Mirtazapine | Dimetacrine | Unconfirmed |
| Ketotifen | Ethanol | Unconfirmed |
| Paraldehyde | Aminocaproic acid | Unconfirmed |
| **Thalidomide** | **Dimetacrine** | **DrugBank** |
| Hydrocodone | Isothipendyl | Unconfirmed |
| Paraldehyde | Tranexamic acid | Unconfirmed |
| Ketotifen | Doxylamine | Unconfirmed |
| Ethanol | Pirenzepine | Unconfirmed |

Table S10.   The validation results for the top 20 predicted DDIs of type “The metabolism of #Drug2 can be increased when combined with #Drug1”.

| **Predicted DDIs** | | **Evidence** |
| --- | --- | --- |
| Nevirapine | Methimazole | Unconfirmed |
| Rifabutin | Methimazole | Unconfirmed |
| **Rifabutin** | **Tazarotene** | **DrugBank** |
| Rifapentine | Methimazole | Unconfirmed |
| **Pentobarbital** | **Doxercalciferol** | **Drugs.com** |
| **Primidone** | **Doxercalciferol** | **Drugs.com** |
| Rifapentine | Nafcillin | Unconfirmed |
| Secobarbital | Primaquine | Unconfirmed |
| **Phenobarbital** | **Methysergide** | **DrugBank** |
| **Pentobarbital** | **Methysergide** | **DrugBank** |
| **Phenobarbital** | **Methylergometrine** | **DrugBank** |
| Primidone | Methimazole | Unconfirmed |
| Nevirapine | Dimethyl sulfoxide | Unconfirmed |
| Phenobarbital | Nafcillin | Unconfirmed |
| **Pentobarbital** | **Prucalopride** | **DrugBank** |
| **Primidone** | **Prucalopride** | **DrugBank** |
| **Primidone** | **Dihydrotachysterol** | **Drugs.com** |
| **Phenobarbital** | **Prucalopride** | **DrugBank** |
| Rifabutin | Dimethyl sulfoxide | Unconfirmed |
| Rifabutin | Prucalopride | Unconfirmed |

Table S11.   The validation results for the top 20 predicted DDIs of type “#Drug1 may decrease the antihypertensive activities of #Drug2”.

| **Predicted DDIs** | | **Evidence** |
| --- | --- | --- |
| Amiloride | Yohimbine | Unconfirmed |
| **Practolol** | **Morniflumate** | **DrugBank** |
| **Eplerenone** | **Yohimbine** | **DrugBank** |
| Naphazoline | Yohimbine | Unconfirmed |
| Hydralazine | Trolamine salicylate | Unconfirmed |
| **Penbutolol** | **Morniflumate** | **DrugBank** |
| Nylidrin | Yohimbine | Unconfirmed |
| Penbutolol | Menadione | Unconfirmed |
| Penbutolol | Methyl salicylate | Unconfirmed |
| **Spironolactone** | **Yohimbine** | **DrugBank** |
| Penbutolol | Fenofibrate | Unconfirmed |
| Apraclonidine | Yohimbine | Unconfirmed |
| Levobunolol | Menadione | Unconfirmed |
| Arotinolol | Menadione | Unconfirmed |
| **Esmolol** | **Morniflumate** | **DrugBank** |
| Pindolol | Menadione | Unconfirmed |
| Alprenolol | Trolamine salicylate | Unconfirmed |
| Bevantolol | Trolamine salicylate | Unconfirmed |
| Alprenolol | Fenofibrate | Unconfirmed |
| Levobunolol | Fenofibrate | Unconfirmed |

Table S12.   The validation results for the top 20 predicted DDIs of type “#Drug1 may increase the anticoagulant activities of #Drug2”.

| **Predicted DDIs** | | **Evidence** |
| --- | --- | --- |
| Phenindione | Mequinol | Unconfirmed |
| Acenocoumarol | Phenylacetic acid | Unconfirmed |
| Warfarin | Acitretin | Unconfirmed |
| Phenindione | Acitretin | Unconfirmed |
| Phenprocoumon | Levosimendan | Unconfirmed |
| Phenprocoumon | Phenylacetic acid | Unconfirmed |
| Citric acid | Aminophenazone | Unconfirmed |
| Acenocoumarol | Levosimendan | Unconfirmed |
| Phenprocoumon | Acitretin | Unconfirmed |
| Acenocoumarol | Acitretin | Unconfirmed |
| Phenindione | Phenylacetic acid | Unconfirmed |
| Phenprocoumon | Mequinol | Unconfirmed |
| **Dicoumarol** | **Cefprozil** | **Drugs.com** |
| **Warfarin** | **Ceftibuten** | **Drugs.com** |
| Phenindione | Doconexent | Unconfirmed |
| Acenocoumarol | Morniflumate | Unconfirmed |
| **Phenindione** | **Bismuth subsalicylate** | **DrugBank** |
| Dicoumarol | Levosimendan | Unconfirmed |
| **Warfarin** | **Metformin** | **Drugs.com** |
| Phenindione | Menadione | Unconfirmed |

Table S13.   The validation results for the top 20 predicted DDIs of type “#Drug1 may increase the hypoglycemic activities of #Drug2”.

| **Predicted DDIs** | | **Evidence** |
| --- | --- | --- |
| Glisoxepide | Oxandrolone | Unconfirmed |
| **Glisoxepide** | **Nandrolone decanoate** | **DrugBank** |
| **Glisoxepide** | **Oxymetholone** | **DrugBank** |
| **Gliquidone** | **Testosterone** | **DrugBank** |
| **Gliquidone** | **Methyltestosterone** | **DrugBank** |
| **Glisoxepide** | **Methyltestosterone** | **DrugBank** |
| Glisoxepide | Tranylcypromine | Unconfirmed |
| **Glisoxepide** | **Testosterone** | **DrugBank** |
| **Glisoxepide** | **Nateglinide** | **DrugBank** |
| **Glisoxepide** | **Testosterone propionate** | **DrugBank** |
| **Glisoxepide** | **Phenelzine** | **DrugBank** |
| Tolazamide | Metamfetamine | Unconfirmed |
| **Gliquidone** | **Selegiline** | **DrugBank** |
| **Gliquidone** | **Oxymetholone** | **DrugBank** |
| **Glisoxepide** | **Chlorpropamide** | **DrugBank** |
| **Glisoxepide** | **Moclobemide** | **DrugBank** |
| **Gliquidone** | **Testosterone propionate** | **DrugBank** |
| Tolazamide | Sulfaphenazole | Unconfirmed |
| Glisoxepide | Pseudoephedrine | Unconfirmed |
| Tolazamide | Dalfampridine | Unconfirmed |

Table S14.   The validation results for the top 20 predicted DDIs of type “#Drug1 may decrease the excretion rate of #Drug2 which could result in a higher serum level”.

| **Predicted DDIs** | | **Evidence** |
| --- | --- | --- |
| Cholic Acid | Zafirlukast | Unconfirmed |
| **Cholic Acid** | **Nelfinavir** | **DrugBank** |
| Cholic Acid | Vismodegib | Unconfirmed |
| Cholic Acid | Cinacalcet | Unconfirmed |
| Cholic Acid | Trimethoprim | Unconfirmed |
| **Amikacin** | **Talniflumate** | **DrugBank** |
| Cholic Acid | Dabrafenib | Unconfirmed |
| Cholic Acid | Sulfinpyrazone | Unconfirmed |
| Cholic Acid | Cabozantinib | Unconfirmed |
| Cholic Acid | Montelukast | Unconfirmed |
| Cholic Acid | Asunaprevir | Unconfirmed |
| **Cholic Acid** | **Lenvatinib** | **DrugBank** |
| Cholic Acid | Carbamazepine | Unconfirmed |
| Cholic Acid | Lobeglitazone | Unconfirmed |
| Cholic Acid | Prasugrel | Unconfirmed |
| Streptozocin | Menadione | Unconfirmed |
| **Streptomycin** | **Talniflumate** | **DrugBank** |
| **Gentamicin** | **Morniflumate** | **DrugBank** |
| Valrubicin | Menadione | Unconfirmed |
| **Cholic Acid** | **Efavirenz** | **DrugBank** |

Table S15.   The validation results for the top 20 predicted DDIs of type “#Drug1 may increase the bradycardic activities of #Drug2”.

| **Predicted DDIs** | | **Evidence** |
| --- | --- | --- |
| **Succinylcholine** | **Sotalol** | **Drugs.com** |
| Bretylium | Vapreotide | Unconfirmed |
| **Succinylcholine** | **Esmolol** | **Drugs.com** |
| **Succinylcholine** | **Levobunolol** | **Drugs.com** |
| Succinylcholine | Arotinolol | Unconfirmed |
| **Succinylcholine** | **Bisoprolol** | **Drugs.com** |
| **Succinylcholine** | **Atenolol** | **Drugs.com** |
| **Ruxolitinib** | **Bevantolol** | **DrugBank** |
| **Ruxolitinib** | **Arotinolol** | **DrugBank** |
| Succinylcholine | Alprenolol | Unconfirmed |
| **Succinylcholine** | **Metoprolol** | **Drugs.com** |
| **Succinylcholine** | **Propranolol** | **Drugs.com** |
| Succinylcholine | Bupranolol | Unconfirmed |
| **Succinylcholine** | **Penbutolol** | **Drugs.com** |
| **Tofacitinib** | **Bevantolol** | **DrugBank** |
| **Succinylcholine** | **Carvedilol** | **Drugs.com** |
| **Edrophonium** | **Ivabradine** | **Drugs.com** |
| **Succinylcholine** | **Pindolol** | **Drugs.com** |
| **Tofacitinib** | **Arotinolol** | **DrugBank** |
| **Pyridostigmine** | **Ivabradine** | **Drugs.com** |

Table S16.   The validation results for the top 20 predicted DDIs of type “#Drug1 may increase the hypokalemic activities of #Drug2”.

| **Predicted DDIs** | | **Evidence** |
| --- | --- | --- |
| **Indacaterol** | **Benzthiazide** | **DrugBank** |
| **Hexoprenaline** | **Torasemide** | **DrugBank** |
| Indacaterol | Levomenol | Unconfirmed |
| **Hexoprenaline** | **Etacrynic acid** | **DrugBank** |
| **Hexoprenaline** | **Chlorothiazide** | **DrugBank** |
| **Hexoprenaline** | **Hydrochlorothiazide** | **DrugBank** |
| **Indacaterol** | **Mannitol** | **Drugs.com** |
| Indacaterol | Amiloride | Unconfirmed |
| Indacaterol | Isosorbide mononitrate | Unconfirmed |
| **Indacaterol** | **Cyclothiazide** | **DrugBank** |
| Indacaterol | Papaverine | Unconfirmed |
| Indacaterol | Aminolevulinic acid | Unconfirmed |
| **Indacaterol** | **Acetazolamide** | **Drugs.com** |
| **Hexoprenaline** | **Quinethazone** | **DrugBank** |
| Indacaterol | Technetium Tc-99m sestamibi | Unconfirmed |
| **Hexoprenaline** | **Furosemide** | **DrugBank** |
| **Hexoprenaline** | **Hydroflumethiazide** | **DrugBank** |
| **Hexoprenaline** | **Polythiazide** | **DrugBank** |
| Indacaterol | Chloroxine | Unconfirmed |
| **Indacaterol** | **Methazolamide** | **Drugs.com** |

**Section C**

| **Algorithm 1** KG feature extraction using TransE and GCN |
| --- |
| **Input:** Knowledge graph $G_{kg}=\left\{\left(h,r,t\right)\vert h,t\in E,r\in R\right\}$, embedding dimension *d*, number of GCN layers *L*, margin hyperparameter $\lambda$.  **Output:** KG feature $s_{KG\_\left(d_{i},d_{j}\right)}\in R^{800\times1}$ of drug pair $\left(d_{i},d_{j}\right)$.  1: **for** each triple $\left(h,r,t\right)$ in $G_{kg}$ **do**  2: **Initialize** $v_{h},v_{r},v_{t}\in R^{d}$ randomly  3: Optimize TransE loss:  4: $L_{TransE}=\sum_{\left(h,r,t\right)\in Q} \sum_{\left(h',r,t'\right)\in Q'} \left[t\left(v_{h},v_{r},v_{t}\right)+\lambda-t\left(v_{h'},v_{r},v_{t'}\right)\right]_{+}$ 5: where $t\left(v_{h},v_{r},v_{t}\right)=\left\Vert v_{h}+v_{r}-v_{t}\right\Vert_{L2}$ 6: **end for**  7: Obtain original embedding $h_{original}\in R^{400\times1}$ of the drug node  8: **Initialize** $h_{i}^{\left(0\right)}=h_{original\_i}$ for each node $i\in E$ 9: **for** *l* = 0 to *L*-1 **do**  10: **for** each node $i\in E$ **do**  11: $h_{i}^{\left(l+1\right)}=\mathrm{ReLU}\left(\sum_{j\in N_{i}} \omega_{ij}W^{\left(l\right)}h_{j}^{\left(l\right)}+\omega_{i}h_{i}^{\left(l\right)}\right)$ 12: where $\omega_{ij}=1/\sqrt{\left\vert N_{i}\right\vert\left\vert N_{j}\right\vert}$, $\omega_{i}=1/\left\vert N_{i}\right\vert$ 13: **end for**  14: **end for**  15: **for** each drug node *d* **do**  16: $s_{KG\_d}=\mathrm{Normalize}(h_{d}^{(L)})$ 17: **end for**  18: **for** each drug pair $\left(d_{i},d_{j}\right)$ **do**  19: $s_{KG\_\left(d_{i},d_{j}\right)}=\mathrm{concatenate}(s_{KG\_d_{i}}, s_{KG\_d_{j}})$ 20: **end for**  21: **return** $s_{KG\_\left(d_{i},d_{j}\right)}\in R^{800\times1}$ |

| **Algorithm 2** Molecular graph feature extraction using EGAT |
| --- |
| **Input:** SMILES sequence of drug *d*.  **Output:** Molecular graph feature $s_{MG\_\left(d_{i},d_{j}\right)}\in R^{1024\times1}$ of drug pair $\left(d_{i},d_{j}\right)$.  1: Parse SMILES sequence to obtain molecular graph  2: **for** each atom node *i* **do**  3: Extract 69-dimensional initial node feature $n_{i}$ 4: **end for**  5: **for** each bond edge $\left(i, j\right)$ **do**  6: Extract 6-dimensional initial edge feature $e_{ij}$ 7: **end for**  8: **for** *l* = 0 to 3 **do**  9: **for** each edge $\left(i, j\right)$ **do**  10: $e'_{ij}=W_{e}\left(n_{i}\vert\vert e_{ij}\vert\vert n_{j}\right)+b_{e}$ 11: **end for**  12: **for** each node *i* **do**  13: $n_{i}^{'}=W_{n}n_{i}+b_{n}$ 14: **end for**  15: **for** each edge $\left(i, j\right)$ **do**  16: Reshape $e'_{ij}$ → $e'_{ij,h}\in R^{128\times1\times4}$ 17: $\varepsilon_{ij}=W \text{LeakyReLU}\left(e'_{ij,h}\right)$ 18: **end for**  19: **for** each node *i* **do**  20: **for** each neighbor $j\in N_{i}$ **do**  21: $\alpha_{ij}=\text{softmax}\left(\varepsilon_{ij}\right)=\frac{\exp\left(\varepsilon_{ij}\right)}{\sum_{k\in N_{i}} \exp\left(\varepsilon_{ik}\right)}$ 22: **end for**  23: **end for**  24: **for** each node *i* **do**  25: Reshape $n'_{i}$ → $n'_{i,h}\in R^{128\times1\times4}$ 26: $n_{i\_new,h}=\sum_{k\in N_{i}} \alpha_{ik}n'_{k,h}$ 27: Reshape $n_{i\_new,h}$ → $n_{i\_new}\in R^{512\times1}$ 28: **end for**  29: $n_{i}=n_{i\_new}$ for each node *i*  30: $e_{ij}=e'_{ij}$ for each edge $\left(i, j\right)$ 31: **end for**  32: $s_{MG\_d}=\text{GlobalAvgPooling}(\left\{n_{i}\right\})$ 33: **for** each drug pair $\left(d_{i},d_{j}\right)$ **do**  34: $s_{MG\_\left(d_{i},d_{j}\right)}=\mathrm{concatenate}(s_{MG\_d_{i}}, s_{MG\_d_{j}})$ 35: **end for**  36: **return** $s_{MG\_\left(d_{i},d_{j}\right)}\in R^{1024\times1}$ |

| **Algorithm 3** Multimodal feature fusion using capsule network |
| --- |
| **Input:** Multi-modal features $s_{modality\_\left(d_{i},d_{j}\right)}=\left\{s_{KG\_\left(d_{i},d_{j}\right)},s_{FP\_\left(d_{i},d_{j}\right)},s_{MG\_\left(d_{i},d_{j}\right)},s_{PH\_\left(d_{i},d_{j}\right)}\right\}$ of drug pair $\left(d_{i},d_{j}\right)$ **Output:** Comprehensive feature representation $S_{\left(d_{i},d_{j}\right)}\in R^{1024\times1}$ of drug pair $\left(d_{i},d_{j}\right)$.  1: **for** each modality feature in $s_{modality}$ **do**  2: $S_{modality}=W_{transform}\cdot s_{modality}$ 3: **end for**  4: **for** each $s_{i}$ in $S_{modality}$ **do**  5: $c_{i}^{0}=Squashing\left(s_{i}\right)=\frac{\left\Vert s_{i}\right\Vert^{2}}{1+\left\Vert s_{i}\right\Vert^{2}}\frac{s_{i}}{\left\Vert s_{i}\right\Vert}$ 6: **end for**  7: $C^{0}=\left\{c_{i}^{0}\right\}$ 8: **for** *l* = 0 to 1 **do**  9: **Initialize** routing logits $m_{ij}^{l}=0$ 10: **for** *r* = 1 to routing iterations number **do**  11: $\beta_{ij}^{l}=\frac{\exp\left(m_{ij}^{l}\right)}{\sum_{k} \exp\left(m_{ik}^{l}\right)}$ 12: **for** each capsule *i* in $C^{l}$ **do**  13: **for** each output capsule *j* **do**  14: $a_{ij}^{l}=W_{ij}^{l}c_{i}^{l}$ 15: **end for**  16: **end for**  17: **for** each output capsule *j* **do**  18: $f_{j}^{l}=\sum_{i} \beta_{ij}^{l}a_{ij}^{l}$ 19: $c_{j}^{l+1}=\mathrm{Squashing}\left(f_{j}^{l}\right)$ 20: **end for**  21: **for** each capsule *i*, *j* **do**  22: $m_{ij}^{l}=m_{ij}^{l}+a_{ij}^{l}c_{j}^{l+1}$ 23: **end for**  24: **end for**  25: Set $C^{l+1}=\left\{c_{j}^{l+1}\right\}$ 26: **end for**  27: $S_{\left(d_{i},d_{j}\right)}=\mathrm{Flatten}(C^{2})$ 28: **return** $S_{\left(d_{i},d_{j}\right)}\in R^{1024\times1}$ |

1. Corresponding author. [↑](#footnote-ref-1)
